# Hipertensão em Indígenas Brasileiros: Uma Crise de Saúde Pública em Rápido Crescimento

**DOI:** 10.36660/abc.20250567

**Published:** 2025-10-28

**Authors:** Allan K. N. Alencar

**Affiliations:** 1 Tulane University Department of Biomedical Engineering New Orleans LA EUA Department of Biomedical Engineering, Tulane University, New Orleans, LA – EUA

**Keywords:** Saúde de Populações Indígenas, Hipertensão, Disparidades nos Níveis de Saúde, Fatores de Risco de Doenças Cardíacas, Transição Epidemiológica

O aumento da carga de hipertensão arterial sistêmica entre as populações indígenas do Brasil é um claro indicador da transição epidemiológica em curso no país, marcada por uma mudança de doenças infecciosas e desnutrição para doenças crônicas não transmissíveis (DCNT), como a hipertensão. No estudo recente de Araújo et al.,^1^ realizado em um ambulatório especializado em saúde indígena em Passo Fundo (RS), 26,0% dos adultos indígenas foram considerados hipertensos. Essa prevalência é consistente com relatos anteriores de outras populações indígenas no Brasil, como as do Parque Indígena do Xingu (26,7%) e da região do Alto Rio Negro (29,0%),^2^ sugerindo que os achados refletem uma tendência nacional mais ampla de aumento do risco cardiovascular entre comunidades indígenas. Vários estudos destacam essa transição, observando que as comunidades indígenas estão enfrentando taxas crescentes de hipertensão e outras condições cardiometabólicas como resultado de rápidas mudanças culturais, econômicas e de estilo de vida, incluindo urbanização, mudanças alimentares e maior interação com a sociedade não indígena.^3–6^ Pesquisas nacionais confirmam o surgimento de obesidade, hipertensão e diabetes em um número crescente de comunidades indígenas em todas as regiões do Brasil, destacando ainda mais a transição epidemiológica.^7,8^ Antes considerada virtualmente ausente nessas comunidades, a hipertensão agora está emergindo como uma preocupação crítica de saúde, com taxas de prevalência se aproximando ou até mesmo superando as médias nacionais.

O estudo de Araújo et al.^1^ acrescenta nuances a esse quadro epidemiológico ao identificar fatores independentemente associados à hipertensão: idade ≥ 60 anos, cônjuge e diabetes mellitus. A constatação de que 58% dos idosos nessa população são hipertensos é especialmente preocupante quando comparada a dados demográficos que mostram que apenas 20,1% dos indígenas no Rio Grande do Sul têm mais de 60 anos.^2^ Isso sugere uma alta carga de doenças entre os idosos e reforça a urgência da implementação de estratégias preventivas específicas para cada idade.

Um aspecto intrigante do estudo é a associação entre estado civil e hipertensão. Contrariamente à literatura epidemiológica convencional, onde o casamento é frequentemente considerado protetor contra doenças cardiovasculares por meio de mecanismos de apoio social,^9^ este estudo constatou que ter um cônjuge estava associado a uma prevalência 1,61 vezes maior de hipertensão. Essa divergência pode refletir pressões socioculturais ou econômicas específicas dentro das famílias indígenas, que merecem uma investigação qualitativa mais aprofundada.

A forte ligação com o diabetes mellitus (razão de prevalência = 2,33) também se alinha ao crescente conjunto de evidências sobre a convergência de distúrbios metabólicos entre populações indígenas.^10–12^ Vias fisiopatológicas compartilhadas, como resistência à insulina e inflamação crônica, podem contribuir para o aumento do risco cardiovascular, particularmente no contexto de acesso precário a cuidados primários culturalmente competentes.

Além dos fatores de risco individuais, os determinantes estruturais e sistêmicos também devem ser abordados. As desigualdades em saúde que afetam as comunidades indígenas no Brasil estão profundamente enraizadas na marginalização histórica, no isolamento geográfico e na escassez de recursos para serviços de saúde.^13^ A Política Nacional de Saúde Integral Indígena (PNASPI) avançou no estabelecimento de modelos diferenciados de atenção, mas ainda existem lacunas na prevenção de doenças crônicas, no rastreamento e na continuidade do cuidado.^14^ O fortalecimento da atenção primária à saúde, por meio de clínicas móveis, agentes comunitários de saúde e na integração de práticas tradicionais de cura, será fundamental para reverter essas tendências.^15^

Os achados de Araújo et al.^1^ reforçam ainda mais a necessidade de estudos longitudinais e comunitários para avaliar a real carga da hipertensão e outras DCNTs em populações indígenas. Pesquisas futuras devem explorar a influência do sódio na dieta, do estresse psicossocial e da degradação ambiental, incluindo ameaças aos sistemas alimentares tradicionais e ao acesso à água, como potenciais contribuintes para o risco cardiovascular.^2^

Em conclusão, a hipertensão não é mais uma exceção, mas uma epidemia iminente entre as populações indígenas do Brasil. O estudo de Araújo et al.^1^ deixa claro: a transição epidemiológica não é uma previsão distante; ela já está em andamento. O que se exige urgentemente é uma resposta nacional coordenada que integre prevenção adaptada à cultura, atendimento clínico baseado em evidências e investimento sustentado em infraestrutura de saúde indígena. Qualquer medida menos grave corre o risco de agravar as desigualdades históricas e prejudicar uma população que não pode mais esperar.

## References

[1] Araújo JM, Tuzzin L, Borges DT, Polettini J, Rabello RS, Acrani GO (2025). Prevalence of Systemic Arterial Hypertension and Associated Factors in Indigenous Treated at a Specialized Outpatient Clinic in Southern Brazil. Arq Bras Cardiol.

[2] Gimeno SG, Rodrigues D, Canó EN, Lima EE, Schaper M, Pagliaro H (2009). Cardiovascular Risk Factors Among Brazilian Karib Indigenous Peoples: Upper Xingu, Central Brazil, 2000-3. J Epidemiol Community Health.

[3] Souza ZA, Ferreira AA, Santos J, Meira KC, Pierin AMG (2018). Cardiovascular Risk Factors with an Emphasis on Hypertension in the Mura Indians from Amazonia. BMC Public Health.

[4] Kramer CK, Leitão CB, Viana LV (2022). The Impact of Urbanisation on the Cardiometabolic Health of Indigenous Brazilian Peoples: A Systematic Review and Meta-Analysis, and Data from the Brazilian Health Registry. Lancet.

[5] Souza ZA, Ferreira AA, Santos BD, Pierin AM (2015). Hypertension Prevalence Among Indigenous Populations in Brazil: A Systematic Review with Meta-Analysis. Rev Esc Enferm USP.

[6] Baniwa EJF, Sacuena ERP, Noce RRD, Quaresma VB, Alencar TH, Lemes RB (2024). Fourteen-Year Trends in Overweight, General Obesity, and Abdominal Obesity in Amazonian Indigenous Peoples. BMC Public Health.

[7] Coimbra CE, Santos RV, Welch JR, Cardoso AM, Souza MC, Garnelo L (2013). The First National Survey of Indigenous People's Health and Nutrition in Brazil: Rationale, Methodology, and Overview of Results. BMC Public Health.

[8] Brasil (2022). Ministério da Saúde. Perfil Demográfico e Epidemiológico dos Povos Indígenas no Brasil.

[9] Robles TF, Kiecolt-Glaser JK (2003). The Physiology of Marriage: Pathways to Health. Physiol Behav.

[10] Mendoza-Caamal EC, Barajas-Olmos F, García-Ortiz H, Cicerón-Arellano I, Martínez-Hernández A, Córdova EJ (2020). Metabolic Syndrome in Indigenous Communities in Mexico: A Descriptive and Cross-Sectional Study. BMC Public Health.

[11] Godfrey TM, Cordova-Marks FM, Jones D, Melton F, Breathett K (2022). Metabolic Syndrome among American Indian and Alaska Native Populations: Implications for Cardiovascular Health. Curr Hypertens Rep.

[12] McKay CD, O’Bryan E, Gubhaju L, McNamara B, Gibberd AJ, Azzopardi P (2022). Potential Determinants of Cardio-Metabolic Risk among Aboriginal and Torres Strait Islander Children and Adolescents: A Systematic Review. Int J Environ Res Public Health.

[13] Montenegro RA, Stephens C (2006). Indigenous Health in Latin America and the Caribbean. Lancet.

[14] Carlos EA (2014). Health and Indigenous Peoples in Brazil: Reflections Based on the First National Survey of Indigenous People's Health and Nutrition. Cad Saude Publica.

[15] Langdon EJ, Wiik FB (2010). Anthropology, Health and Illness: An Introduction to the Concept of Culture Applied to the Health Sciences. Rev Lat Am Enfermagem.

