# Supplementary material for: Hipertensão em Indígenas Brasileiros: Uma Crise de Saúde Pública em Rápido Crescimento
Source: Arq Bras Cardiol. 2025 Oct 28;122(9):e20250567. [Article in Portuguese] doi: 10.36660/abc.20250567 (PMC12671717; doi:10.36660/abc.20250567)
Supplement: Translation

# Translation: Hypertension in Indigenous Brazilians: A Rapidly Emerging Public Health Crisis

**DOI:** 10.36660/abc.20250567i

**Authors:** Allan K. N. Alencar

**Affiliations:** 1 New Orleans LA EUA Department of Biomedical Engineering, Tulane University, New Orleans, LA – EUA

**Keywords:** Health of Indigenous Peoples, Hypertension, Health Status Disparities, Heart Disease Risk Factors, Health Transition

The increasing burden of systemic arterial hypertension among Brazil's Indigenous populations is a clear indicator of the country's ongoing epidemiological transition, marked by a shift from infectious diseases and malnutrition to non-communicable chronic diseases (NCDs) such as hypertension. In the recent study by Araújo et al.,^1^ conducted in a specialized outpatient clinic for Indigenous health in Passo Fundo (RS), 26.0% of Indigenous adults were found to be hypertensive. This prevalence is consistent with previous reports from other Indigenous populations in Brazil, such as those from the Xingu Indigenous Park (26.7%) and the Upper Rio Negro region (29.0%),^2^ suggesting that the findings reflect a broader national trend of increasing cardiovascular risk among Indigenous communities. Multiple studies highlight this transition, noting that Indigenous communities are experiencing rising rates of hypertension and other cardiometabolic conditions as a result of rapid cultural, economic, and lifestyle changes, including urbanization, dietary shifts, and increased interaction with non-Indigenous society.^3–6^ National surveys confirm the emergence of obesity, hypertension, and diabetes in a growing number of Indigenous communities across all regions of Brazil, further underscoring the epidemiological transition.^7,8^ Once considered virtually absent in these communities, hypertension is now emerging as a critical health concern, with prevalence rates approaching or even surpassing national averages.

The study by Araújo et al.^1^ adds nuance to this epidemiological picture by identifying factors independently associated with hypertension: age ≥60 years, having a spouse, and diabetes mellitus. The finding that 58% of older individuals in this population are hypertensive is especially troubling when juxtaposed with demographic data showing that only 20.1% of Indigenous people in Rio Grande do Sul are over 60 years old.^2^ This suggests a high disease burden among the elderly and reinforces the urgency of implementing age-specific preventive strategies.

An intriguing aspect of the study is the association between marital status and hypertension. Contrary to conventional epidemiological literature, where marriage is often considered protective against cardiovascular disease via social support mechanisms,^9^ this study found that having a spouse was associated with a 1.61-fold higher prevalence of hypertension. This divergence may reflect unique sociocultural or economic pressures within Indigenous households that merit further qualitative investigation.

The strong link with diabetes mellitus (prevalence ratio = 2.33) also aligns with the growing body of evidence on the convergence of metabolic disorders among Indigenous populations.^10–12^ Shared pathophysiological pathways, such as insulin resistance and chronic inflammation, may contribute to the heightened cardiovascular risk, particularly in the context of poor access to culturally competent primary care.

Beyond individual risk factors, structural and systemic determinants must also be addressed. Health inequalities affecting Indigenous communities in Brazil are deeply rooted in historical marginalization, geographic isolation, and under-resourced health services.^13^ The National Policy for Comprehensive Indigenous Health (PNASPI) has made strides in establishing differentiated care models, yet gaps remain in chronic disease prevention, screening, and continuity of care.^14^ Strengthening primary healthcare, through mobile clinics, community health agents, and integration of traditional healing practices, will be key to reversing these trends.^15^

The findings by Araújo et al.^1^ further support the call for longitudinal and community-based studies to assess the true burden of hypertension and other NCDs in Indigenous populations. Future research should explore the influence of dietary sodium, psychosocial stress, and environmental degradation, including threats to traditional food systems and water access, as potential contributors to cardiovascular risk.^2^

In conclusion, hypertension is no longer an exception but a looming epidemic within Brazil's Indigenous populations. The study by Araújo et al.^1^ makes it unmistakably clear: the epidemiological transition is not a distant forecast; it is already unfolding. What is now urgently required is a coordinated national response that integrates culturally tailored prevention, evidence-based clinical care, and sustained investment in Indigenous health infrastructure. Anything less risks deepening historical inequities and failing a population that can no longer wait.
